# Maxillary Prosthetics, Speech Impairment, and Presidential Politics: How Grover Cleveland Was Able to Speak Normally after His “Secret” Operation

**DOI:** 10.1055/s-0039-3400537

**Published:** 2019-12-02

**Authors:** Margaret Murray, Theodore N. Pappas, David B. Powers

**Affiliations:** 1 Department of Family and Community Medicine, East Virginia Medical School, Norfolk Virginia; 2 Department of Surgery, Duke University School of Medicine, Durham, North Carolina; 3 Division of Craniomaxillofacial Trauma and Reconstructive Surgery, Department of Surgery, Duke University School of Medicine, Durham, North Carolina

**Keywords:** Grover Cleveland, Kasson Gibson, oral surgery, maxillary resection

## Abstract

In the summer of 1893, President Grover Cleveland discovered a mass on the roof of his mouth. Two physicians examined it, determined that it was a neoplasm, and recommended resection. In an effort to avoid revealing the illness to the public, the President and his doctors boarded a yacht on July 1 1893, where the surgeons resected the affected portion of his maxilla and several teeth under an ether anesthetic. Afterward, Kasson C. Gibson, a New York dentist, created a rubber obturator, which was placed in the surgical defect in the maxilla and restored the President's facial contour and speech. Due to the precise reconstruction with the rubber appliance crafted by Gibson, the President lived the rest of his public life without facial or speech abnormality. This article will review the details of the work of Kasson Gibson and the President's maxillary prosthesis.

In the summer of 1893, President Grover Cleveland was embroiled in a controversial debate with Congress over the United States (US) economy. The country was suffering from a financial crisis and the President was attempting to strengthen the financial outlook by repealing the Sherman Silver Purchase Act, which made silver the standard of the US currency. He wanted to return to the gold standard to reestablish confidence in the economy. As Cleveland was preparing to address Congress that summer, he discovered a mass on the roof of his mouth. Two physicians examined it, determined that it was a neoplasm, and recommended resection. In an effort to avoid revealing the illness to the public, the President and his doctors boarded a yacht on July 1 1893, where the surgeons resected the affected portion of his maxilla and several teeth under an ether anesthetic. Afterward, Kasson C. Gibson, a New York dentist, created a rubber obturator, which was placed in the surgical defect in the maxilla and restored the President's facial contour and speech. Cleveland addressed Congress as he was recovering from surgery and no one suspected that the President had undergone a surgical resection of his maxilla. Due to the precise reconstruction with the rubber appliance crafted by Gibson, the President lived the rest of his public life without facial or speech abnormality. Twenty years later, several of the involved surgeons told the world about the secret operation and the perfect reconstructive appliance.

## President Grover Cleveland

Cleveland was born in New Jersey in 1837. After a sparse education, he took a clerical job with his uncle's law firm in Buffalo, New York, where he eventually became a law clerk, read the law, and was admitted to the New York bar in 1859. As a Democrat, he was elected as Sheriff of Erie County, New York in 1871 and Mayor of Buffalo in 1881.

A year later in 1882, he was elected governor of New York and was the Democratic presidential nominee in 1884. In November of 1884, Cleveland defeated the Republican candidate, James G. Blaine, in a narrow election, capping Cleveland's meteoric rise to power.

After an eventful 4-year term, Cleveland ran again this time against the Republican candidate, Benjamin Harrison. Cleveland narrowly won the popular vote but lost the Electoral College. Cleveland returned to his legal practice during the Harrison presidency but in 1892 reemerged to run for the presidency again. This election pitted Cleveland, the Democrat, versus Harrison, the Republican, and included a third-party candidate James Weaver. Cleveland won the popular vote and the Electoral College, becoming the only president to serve two, nonconsecutive, 4-year terms. 1

## The Operations

Two months into his second term, Cleveland noticed a nonpainful mass on the roof of his mouth. On June 18, 1893, Dr. Robert M O'Reilly, the President's personal friend and physician, examined his mouth and found “an ulcerative surface nearly as large as a quarter, with cauliflower granulations, and crater edges with at least one sinus extending to the bone. 2 He determined it was likely a malignant epithelioma. 3 On June 25 ^th^ Dr. Joseph Bryant examined the oval ulcer, “extending from the maxillary molars to the within a quarter of an inch of the midline of the mouth and encroaching on the anterior part of the soft palate.” 4 Bryant, a renowned surgeon at Bellevue Hospital in New York City, had a close relationship with Cleveland that dated back to the early 1880s. A pathologist, Dr. William Welch, at Johns Hopkins, determined the mass to be malignant, 4 and Bryant recommended resection of the mass.

During that summer, the President was managing a financial crisis. It was his belief that the US economy would be more stable if the Sherman Silver Act was repealed and the US dollar was backed by gold. He thought that it was an inopportune time to tell the public and Congress about his need for a cancer operation on his maxilla. Therefore, the President decided to have an operation done secretly on aboard the Oneida, a yacht in the New York Harbor. 4 The ship belonged to Commodore Elias Benedict, a close friend of the President. 4 5 The choice of the yacht, rather than a hospital, was a suitable site for the operation since it was privately owned and had large, well-lit, rooms below deck.

On June 30, a party of prominent physicians, dentists, and surgeons from Bellevue Hospital and Jefferson Medical College assembled on the yacht. The group included Drs. William Keen, a neurological pathologist and brain surgeon, Edward Janeway, an internist, John F Erdmann, a surgeon, and Ferdinand Hasbrouck, a dentist, and anesthesiologist. 6 7 8 9 Later that day the President and Dr. Bryant joined the medical party on the Oneida anchored offshore Manhattan. 10

The operation began at 12:50 pm the following day as Dr. Hasbrouck injected cocaine solution around the tumor and administered nitric oxide to manage the pain. 10 11 Dr. Hasbrouck removed several teeth before Dr. Bryant made an incision around the tumor with an electric carving knife. The anesthetic was deepened with ether and Bryant proceeded to remove the left maxilla, with minimal bleeding. 11 The left cheek was pulled away, and “the left upper jaw was then chiseled loose from the front of the first bicuspid to the posterior extremity of the bone.” 11 A ronguer was used to divide and excise three more teeth and the affected hard palate from the alveolar border to the median line, exposing the maxillary antrum filled with a “gelatinous mass... totally different in appearance from the typical epithelioma of the roof of the mouth” as noted by Dr. Keen. The “mass” was scooped out, exposing the unaffected infraorbital plate. The excised five teeth, third of the upper palate, and piece of upper left jawbone were placed in a jar for later pathologic evaluation. 11

The operation ended at 1:55pm and the President had experienced minimal blood loss and maintained stable vital signs. 10 Postoperatively Keen noted that “with the packing the President's speech was labored but intelligible; without the packing it was resembling the worst imaginable case of cleft palate.” 11 On July 5th the Oneida docked at Gray Gables, the President's vacation home, where he disembarked unassisted. 11

On July 8th Attorney General, Richard Olney, visited Cleveland at Gray Gables. The press had been told that the President simply had some teeth extracted on board the Oneida. Olney recounted that Cleveland “had changed a great deal in appearance, lost a great deal of flesh, and his mouth was so stuffed with antiseptic wads that he could hardly articulate.” The first utterance that I understood was something like this: ‘My God, Olney, they nearly killed me.’ He did not talk much, was very depressed, and at that time acted, and I believe he felt, as if he did not expect to recover.’ 12

About 2 weeks later Dr. Bryant visited the President, inspected the incision site, and determined that the margins of the resection site looked malignant. He summoned the surgical team and on July 17 they reassembled on the Oneida and Bryant resected the presumed positive margin. 10 The operation was brief and the President recovered quickly. 11 The next day the medical team deboarded and President returned to Gray Gables. Five days later, a Treasury Department official visited the President at his vacation home to deliver statistics on the US economy. The official later wrote in his diary of the condition of the President, noting that the “President appear not well at all. [He] had his mouth evidently packed with some kind of bandage – could not speak distinctly. Seemed to me to have some serious trouble with his mouth – looked thoroughly tired out.” 13

## Recovery

The President recovered at Gray Gables while an obturator was created to manage his palatal defect. Following resection of the palate, abnormalities in the President's speech and resonance were expected unless the palate was reconstructed. Resections of the hard palate and portions of the maxilla, as experienced by Cleveland, would normally result in deterioration in word and sentence intelligibility and hyper-nasal speech. Reconstruction with an obturator would hopefully return these speech characteristics near normal. 14 Dr. Kasson Church Gibson, a New York dentist, was asked to fashion a palate obturator to reconstruct the contour of the President's face and allow him to speak clearly. Dr. Gibson had set up a temporary dental laboratory at Gray Gables, where he was able to create a plaster cast of the President's mouth. 15 Based on that cast, he created a palatal obturator made of vulcanized rubber. Gibson explained that that it “was made without teeth, gold clasping the cuspid tooth on the left, second bicuspid and molar on the right, bridging across the opening, with a thick round edge where it came in contact with the cheek.” 15 As Dr. Keen observed, “This supported the cheek in its natural position and prevented it from falling in. When it was in place the President's speech was excellent, even its quality, not being altered.” 11

Since Cleveland was the sitting US president, there could be no facial scaring after the surgery to not rouse public suspicion. Keen noted that the “normal appearance of the eye, the normal voice, and especially the absence of any external scar, which was the most important evidence of all, greatly aided in keeping the operation an entire secret. 11 ” Additionally, the surgeons preserved Cleveland's prominent moustache, taking extra precaution to keep the operation secret.

The prosthesis enabled the reconstruction of Cleveland's original speech quality and facial contour so much so that the public was not aware that the President had undergone an extensive oral surgical operation. The President praised Dr. Gibson in October of that same year when the doctor sent him a new prosthesis. Cleveland thanked him and reported that he wore it “all day with utmost ease and comfort without a shred of packing of any kind.” He also noted that, “[my] wife says that my voice and articulation are much better than they have been for a number of days.” 16 Some months after the operation, the President suffered an earache and was seen by a physician that did not know about the secret operation. The obturator fit so perfectly that the physician assumed it was simply a plate supporting false teeth. 16

## Initial Public Appearance after Operation

On August 4, President Cleveland returned to Washington to deliver a speech to Congress, advocating for the repeal of the Silver Purchase Act. The President was described by reporters to be “well-tanned,” “in perfect health,” “looking well and not the least weary,” even though it was approximately only 2 weeks after the second operation. 17 18 On Monday August 8, Congress convened and received the President's message. Cleveland had two clerks from each chamber read his address, as was tradition at the time. 19 Congress ultimately agreed with Cleveland and voted to repeal the Silver Purchase Act. During these initial public appearances, there had been no suspicion that the President had undergone a major oral surgical operation.

## Exposure of the Operation

Dr. Ferdinand Hasbrouck, who had assisted with the President's initial operation, was the first individual to leak the news of the President's operation to the press. The story of Cleveland's operation was published in the Philadelphia Press on August 29, 1893, a day after the repeal of the Silver Purchase Act. That same evening, Dr. Bryant denied the claim that Cleveland had suffered a tumor, stating: “The President had some teeth pulled last July…,” The New York Times wrote a story denying the claim that the President was a “desperate invalid.” 20 They reported that a federal officer had seen the President a few days ago and he “was looking extremely well, being brown from exposure, cheerful, and happy, and if he had lost either his jawbone or his backbone, the loss of the one did not destroy his appetite nor the loss of the other prevent him from reaching some important conclusions calling for thought and action.” It also cited that on June 20th, the President had dined with a friend, who stated “that Mr. Cleveland was in unusually good spirits. If he had been suffering with a disease of the jaw I am sure I should not have failed to discover some trace of it. Not once did he refer to any physical ailment.” Another cabinet member who saw the President after he returned from his trip to Washington claimed it was impossible for him to have undergone such an operation, concluding, “I never saw him looking better.” 20

On August 30 ^th^ , Cleveland left for Gray Gables and stopped in New York to demonstrate his strength, walking from the ferry terminal to the train station instead of traveling by carriage, his typical mode of transportation. According to the *New York Time* s report, “The President seems a trifle thinner than he was a year ago, but on his face is a ruddy glow. His carriage erect and his actions bespoke a person enjoying perfect health… His face and hands were well browned by his outdoor exercise at Buzzards Bay.” 20 The *World* newspaper described that, “A bright light was falling upon Mr. Cleveland's face for more than ten minutes and every feature was clear and distinct. There was no sign of any operation. There was no swelling, no depression. When he spoke he uttered his words clearly and distinctly, unlike a man part of whose jaw bone had been removed. His eyes were bright and clear and he seemed cheerful and contented.” 21

The next day, Cleveland, his wife, his daughter, and Dr. Bryant departed for Washington and the President “did not look like a very sick man as he sat in the car chatting with the doctor before the train pulled out,” according to the Chicago Daily Tribune. 22 On September 1 of that year, Dr. Bryant declared that the President was “all healed.” 10

## Long-Term Outcome

Cleveland continued to make public speeches and appearances during the remainder of his 3 years of his second term in office. On September 19, 1893, Cleveland appeared at the Centenary of the Foundation of Washington, D.C. and a journalist noted that Cleveland's speech “removed every lingering doubt of his entire soundness of body.” 3 The following January he made a public appearance on New Year's Day and stood for 4 hours, shaking hands with no evidence of trouble. 3 In May, he attended and spoke at the dedication of a monument to Mary Washington, mother of President George Washington, in Fredericksburg, VA. 23

**Fig. 1:**
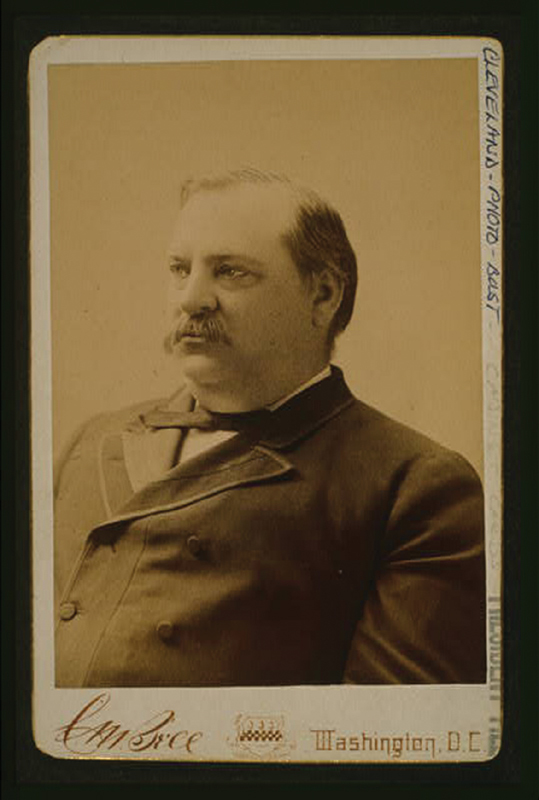
A photograph of President Cleveland in 1888 from the Library of Congress http://loc.gov/pictures/resource/cph.3f06237/ .

**Fig. 2:**
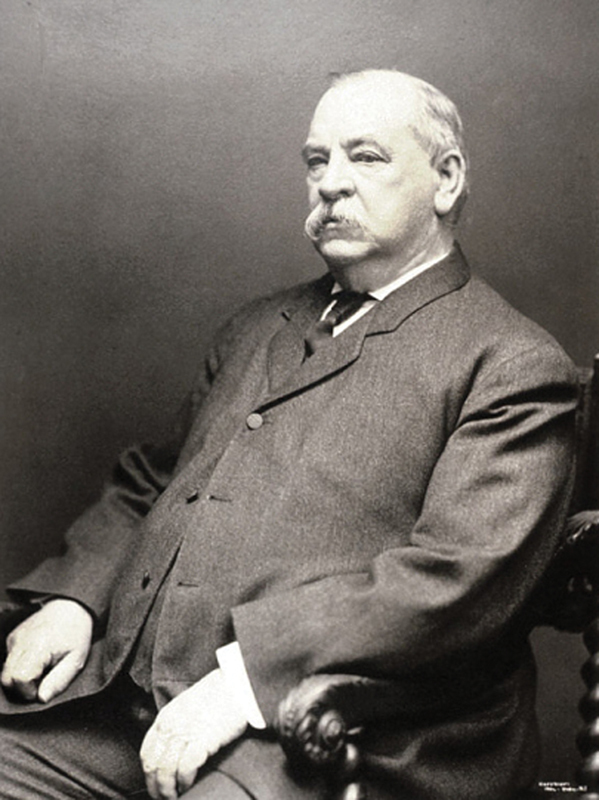
A photograph of President Cleveland in 1904 from the National Portrait Gallery Smithsonian Institute. https://www.si.edu/sisearch?edan_q=grover%2Bcleveland .

In addition to Cleveland's speech, the palate prosthesis maintained the normal contour of the President's face. Fig. 1 shows Cleveland in 1888 demonstrating normal facial contour on his left side. Fig. 2 is a picture from later in life (1904) after his operations and after his second presidency. The left side of his face shows no evidence of any deformity suggesting that his maxilla was resected.

The maxillary obturator functioned well throughout the President's life. The original obturator served as a temporary device for the first several months as noted in a letter from the President to Dr. Gibson dated October 14, 1893. In the letter Cleveland commented on a new obturator (“new plate”), which had arrived at the White House the day before, stating, “I have worn it all day with the utmost ease and comfort…” 24

**Fig. 3:**
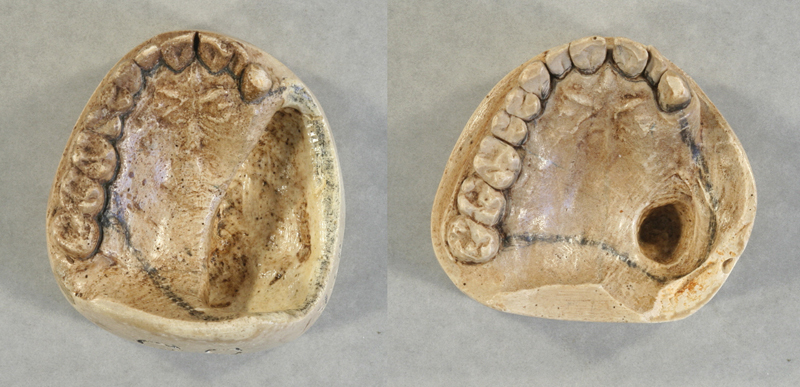
On the left is the cast of the original maxillary defect (1893) and the outline of the size of the obturator that Dr. Gibson crafted. The picture on the right shows a cast of the president's palate from 1897, showing that the defect had contracted over time. Photographs used with permission of the New York Academy of Medicine Archive.

The President's wound continued to heal so much so that in 1897 Dr. Gibson created another cast of his mouth, which indicated that the wound had shrunk from 63.5-by-20.6 mm to 17.5-by 11.1 mm in 4 years since the operation. 15 Fig. 3 shows the plaster casts of Cleveland's maxilla and teeth that Gibson created in 1893 and 1897. The cast taken in 1897 followed a communication from the President suggesting that a small gold clasp had become dislodged from the plate and perhaps another modification was required. 24

## Histology of Cleveland's Tumor

The histology of the Cleveland palatal tumor had been debated for 80 years after the resection. When Dr. Keen examined the President for the first time, he made the clinical diagnosis of epithelioma. Dr O'Reilly biopsied the lesion on June 19th 1893 and the lesion was histologically reviewed by several pathologist, most famously by William Welsh of Johns Hopkins. Dr Welsh thought it was an epithelioma. After resection, the lesion again was reviewed by several pathologists including Dr Welsh. The final diagnosis was carcinoma. Interestingly after the pathologic examination, Dr Keen still referred to the lesion as a sarcoma. Other participants from the operation called it an epithelial cancer, epithelioma, or simply a carcinoma. 11 12 25

Fortunately, all controversies were put to rest in 1980 when pathologists from the University of Pennsylvania asked the Mutter Museum if the specimen could be reexamined grossly and histologically. The gross specimen had been kept in alcohol by Gibson and Keen until it was donated to the Mutter in 1917. After permission was granted, John J. Brooks and his coauthors, from the University of Pennsylvania, grossly examined the specimen and also took biopsies of the 80-year-old tissue and evaluated it by hematoxylin and eosin and a variety of other stains. After gross and microscopic examination plus review of X-rays of the specimen, the lesion was determined to be a verrucous carcinoma likely cured by the initial resection. These authors believe that the clinical course before and after surgery support this histologic diagnosis. 26

## Drs. Bryant and Gibson

Dr. Joseph Decatur Bryant was Cleveland's friend and personal physician. He received his medical degree from Bellevue Hospital Medical College in 1868. He enjoyed a long career on the faculty of Bellevue, achieving the title of Professor of the Principles and Practice of Surgery in 1898. He had one of the largest private surgical practices in New York City while serving as president of the New York Academy of Medicine from 1895 to 1897 and the American Medical Association from 1907 to 1908. Bryant authored one of the leading operative textbooks of his day, *Manual of Operative Surgery* , in 1884 and published on a variety of surgical topics including reporting on the world's largest series of maxillary resection in 1895. 27 28

**Fig. 4:**
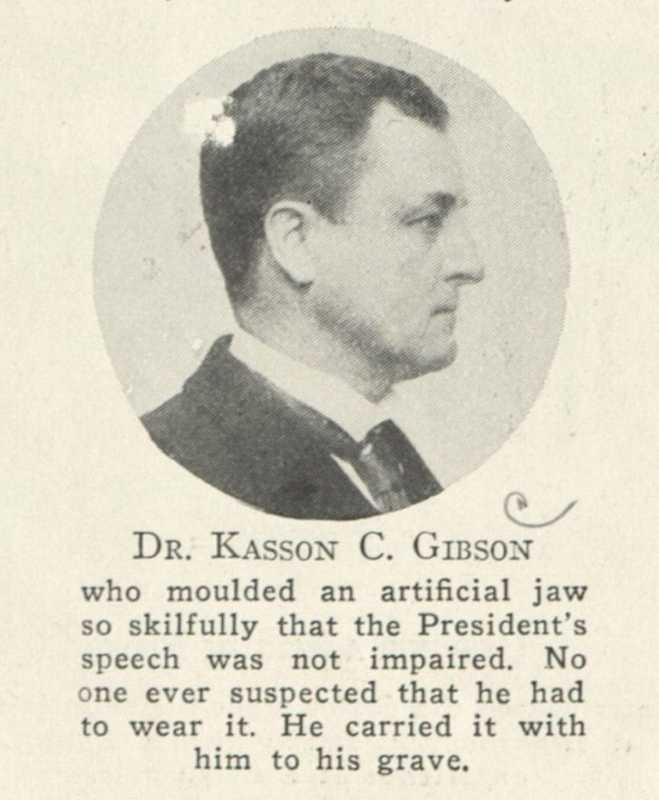
A picture of Kasson Church Gibson (date unknown), courtesy of the New York Academy of Medicine.

**Fig. 5:**
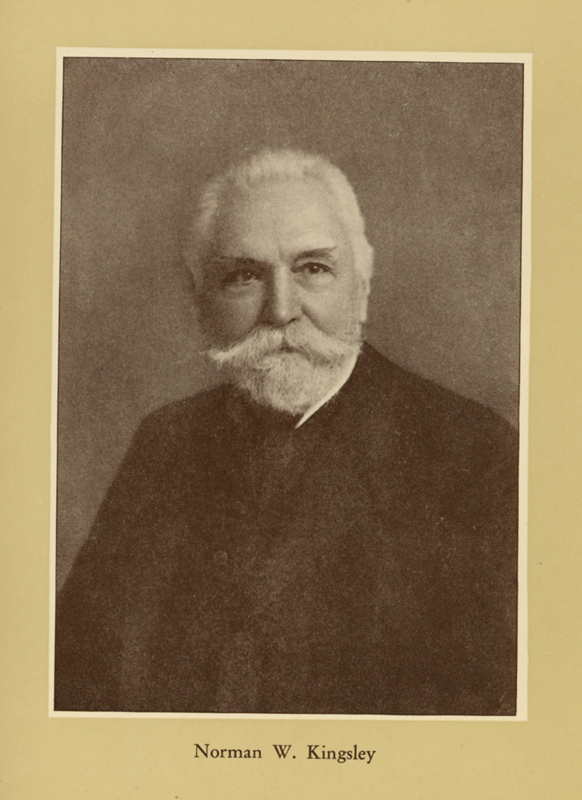
A photograph of Norman William Kingsley, the first Dean of the New York College of Dentistry, ca. 1900. https://doi.org/10.6083/M4VX0F25 .

Kasson Church Gibson ( Fig. 4 ) was born in New South Berlin, NY, in 1849 and was raised in a medical family, as his father was a physician. He left home at the age of 14 years to study in the office of a physician, Joseph Summer of Norwich, NY. He probably knew Joseph Bryant as a youth since Bryant was raised in Norwich and was just 4 years his senior. Both men ended up in health careers and collaborated throughout their professional lives. Gibson moved to New York City in his twenties to study for 5 years in the office of Norman W. Kingsley ( Fig. 5 ), the “father” of the field of orthodontics. 29 Gibson's time with Kingsley was transformational for Gibson. 30

Kingsley was the first to report the use of soft vulcanized Indian rubber as an obturator in a patient with cleft palate. 29 Kingsley also founded the New York College of Dentistry and served as the first Dean from 1865 to 1869. He wrote the first textbook on orthodontics titled *A Treatise on Oral Deformities as a Branch of Mechanical Surgery* . 31 While Kasson Gibson worked in Kingsley's private office, he also attended the College of Dentistry. 30

After Gibson completed his training, he settled into a practice of dentistry in New York City and often collaborated on cases with Bryant. Bryant had a large practice of head and neck surgery, which entailed resecting tumors that resulted in mandibular and maxillary deformities. Bryant would refer his patients to Gibson, who would craft oral prostheses. Therefore, after Bryant removed Cleveland's maxillary tumor, Kasson Gibson was the obvious choice to create the palatal obturator since he had done so many times for Bryant's patients in the past.

Kasson Gibson was clearly the authority in the field when it came to treatment of mandibular and maxillary deformities. Gibson made several important contributions to the dental literature including a report on the correction of oral deformities in his treatise published in the International Dental Journal in 1890. 32 He had a distinguished academic career, serving on the faculty of the Maryland School of Dentistry as a Professor of Oral Deformities and Fractured Maxillaries. 33 He was the treasurer of the Dental Society of the State of New York in 1891 and an active contributor to Dental Cosmos, a popular journal for dentists, and oral surgeons of that era. He died suddenly at home on December 26, 1925. 30

## Conclusion

President Grover Cleveland had a clandestine resection of a maxillary tumor in July, 1893. The resection left the president with a large defect in his palate. The President's dental provider, Kasson C. Gibson, created a rubber obturator that perfectly reconstructed Cleveland's face and allowed for normal speech. Due to the expert work of Gibson, the secrecy around the operation was preserved and allowed the President to function normally throughout the rest of his professional and personal life.
